# Echogenic Content in the Fetal Gallbladder: Systematic Review of Ultrasound Features and Clinical Outcome

**DOI:** 10.3390/diagnostics13020230

**Published:** 2023-01-08

**Authors:** Dan Boitor-Borza, Ioana Cristina Rotar, Adelina Staicu, Roxana Constantin, Daniel Muresan

**Affiliations:** 1 Department of Obstetrics and Gynecology, “Iuliu Hatieganu” University of Medicine and Pharmacy Cluj-Napoca, 400012 Cluj-Napoca, Romania; 2 1st Department of Obstetrics and Gynecology, Emergency County Hospital Cluj-Napoca, 400006 Cluj-Napoca, Romania

**Keywords:** echogenic content, fetal gallbladder, prenatal diagnosis, fetal gallstones, fetal cholelithiasis

## Abstract

It is rare to detect echogenic content in the fetal gallbladder. The etiology, natural course, and prognosis of this condition remain unclear. In addition to providing a systematic review of this topic, we suggest a plan for patient follow-up. From a total of 100 database entries identified in PubMed, EMBASE, and ICTRP reviews, we selected 34 studies in which we investigated the ultrasound features and outcome of this condition. There were 226 fetuses with gallbladder echogenic content identified. Seventy-two fetuses were found to have biliary sludge; thirty cases had a single hyperechogenic focus, and one hundred fetuses had multiple foci in the gallbladder. There were 16 cases of distal shadowing, 37 fetuses with comet tail and twinkling, and 26 cases with no acoustic artifacts. Nine cases of spontaneous resolution before birth have been documented; nine fetuses exhibited no echogenic content at birth, and 138 cases of resolution of echogenic content within the first year of life have been described. Typically, the condition resolves spontaneously during the postnatal period. After adequately reassuring the parents, the patients should be monitored for spontaneous resolution; medical or surgical intervention is not indicated. Asymptomatic patients can be managed with a wait-and-see strategy.

## 1. Introduction

The detection of echogenic content in the fetal gallbladder (EC) intrigues both medical professionals and parents. It may appear as hyperechogenic images or homogenous echogenic material filling the gallbladder (sludge). Authors agree that this condition is still an enigma since little is known about its etiology, natural course, and outcome [1,2,3]. In addition, there are no reports of pathologic analysis of the EC in fetuses [1], and therefore its organic origin cannot be validated.

The fetal normal gallbladder appears on US as an anechoic, long-shaped image with a thin wall. On the visceral face of the liver, it is located ventrally. The fetal gallbladder is typically situated to the right of the umbilical vein, where it may be easily identified, as it shows no flow during a Doppler US. After 30 weeks, its dimensions are between 23 and 26 mm in length and 6 to 8 mm in width, remaining stable until term [3].

In 1983, Beretsky and Lankin described the first case detected incidentally by ultrasound (US) in a woman with preeclampsia [4]. Since then, nearly 40 years have passed and, to the best of our knowledge, only three prospective studies including 54 patients [5,6,7] have been published. The EC is regarded as benign and transient in fetuses, and the prognosis is favorable [2,3,7]. Nevertheless, cases of persistent or complicated EC have been reported; therefore, this assertion is not always accurate. In the absence of prospective cohort studies, many features of EC, such as etiopathogenesis, natural evolution, and outcome, are still unclear.

This article provides a systematic review of the current literature on EC in the fetal gallbladder in order to draw conclusions regarding the evolution and prognosis of this condition. In addition, we suggest an algorithm for the follow-up of concerned patients.

## 2. Materials and Methods

We conducted a multi-database literature review on the subject, focusing on reports of EC identified in fetuses that describe the US diagnosis and outcome. The issues of US features of EC in the fetal gallbladder and clinical outcome is the reason for using prenatally diagnosed EC as a rigorous inclusion criterion.

The protocol for the systematic review and meta-analysis was written in accordance with the Preferred Reporting Items for Systematic Reviews and Meta-Analyses (PRISMA) statement [8] and the international prospective register of systematic reviews (PROSPERO). The registration number is not accessible at this time due to significant delays in protocol publishing, acknowledged by the National Institute of Health Research (NIHR) in the United Kingdom.

We performed a systematic search using the PubMed (MEDLINE) and Cochrane review databases between 1 January 1980 and 1 November 2022, with no restrictions for study design. The search strategy for PubMed was converted to an exact corresponding match for Cochrane, EMBASE and ICTRP reviews and included the following keywords: “fetal/prenatal/antenatal gallbladder” combined with any of the following words: “sludge”, “gallstones”, “cholelithiasis”, “echogenic content”, and “echogenic material”. Bibliographies of all included articles were reviewed for other relevant articles.

Following a formal search, all query entries were collected into bibliography software (Zotero), which was used to remove duplicates based on the title, author(s), and journal names. Using automated open-source software (www.rayyan.ai (accessed on 23 November 2022)), all search results (abstracts and then full-text articles) were assessed for relevance based on the PICOS criteria, which were the inclusion of original case reports or case series (study design) of pregnancies showing EC in the fetal gallbladder (population) that were diagnosed, treated, and followed up in specialized care units (intervention) with available results (outcome).

**Figure 1:**
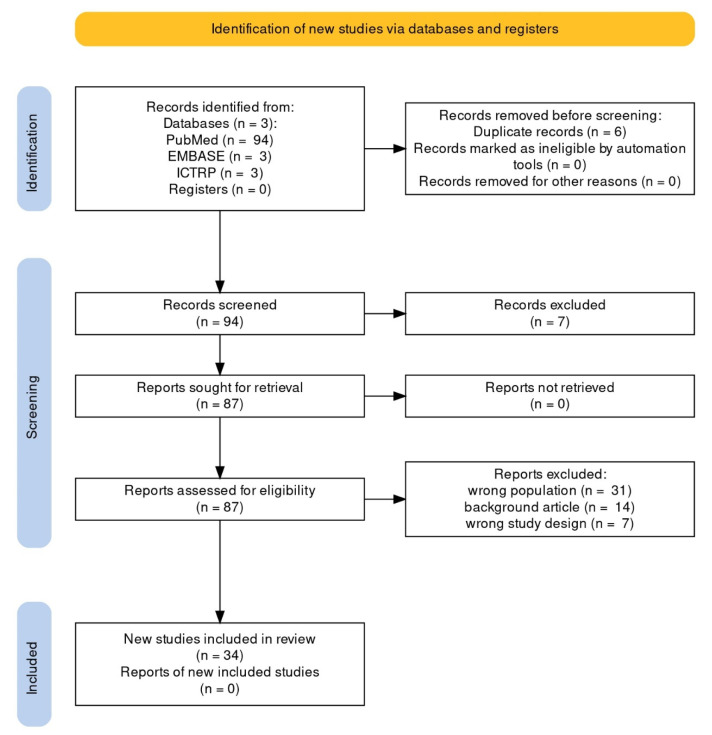
PRISMA flowchart of systematic review article selection process.

Using the search method, a total of 100 articles were retrieved: 94 PubMed reviews and 6 Cochrane reviews (from EMBASE: 3, from ICTRP: 3). We included all English and Spanish-language original papers reporting observed cases of EC; seven articles written in other languages were excluded. Thirty-four articles were included in the qualitative synthesis after removal of duplicates, screening, and PICOS-based evaluation of full texts. Figure 1 depicts the flowchart of the selection procedure.

## 3. Results

Table 1 provides a synthesis of the studies evaluated in the present paper. Thirty-four articles published from 1983 to 2022 were included, reporting on a total of 226 fetuses with EC. We identified twenty-six case reports, six retrospective studies, and three prospective studies, as Brown et al.’s study [5] is divided into prospective and retrospective sections. Twelve articles used the term “gallstones” in the title or abstract, while fourteen employed “cholelithiasis”, five used “echogenic material”, one used “sludge”, one “calcifications”, and one “echogenicities”. Out of a total of 25,777 reported scans, 123 fetuses with EC were identified, equating to an incidence of 0.48%.

The US characteristics of the EC in the fetal gallbladder are reported in Table 2. Seventy-two fetuses were found to have biliary sludge; thirty cases had a single hyperechogenic focus in the gallbladder and one hundred had multiple foci. As for the US artifacts, there were 16 cases of shadowing, 37 fetuses with comet tail and twinkling, and 26 cases with no acoustic artifacts.

In Table 3, the outcomes of newborns with prenatally identified EC in the gallbladder are described. Nine cases of spontaneous resolution before birth have been documented; nine fetuses exhibited no EC at birth, and one hundred and thirty-eight cases of resolution of EC within the first year of life have been described. Six cases of persistent EC after one year of life, including one with symptoms, are explicitly reported.

## 4. Discussion

The objective of this paper is to provide a systematic review of the current literature on EC in the fetal gallbladder. We reviewed 34 studies involving 226 fetuses with biliary EC, from which we can draw certain conclusions regarding its evolution and outcome. Meanwhile, we must admit that the etiopathogenesis of this condition remains an enigma. In addition, we suggest a management algorithm for fetuses with EC in the gallbladder.

### 4.1. Incidence

The vast majority of publications assert that the incidence of EC is very uncommon [1,7,14]. Two retrospective studies report incidences of 0.45% [1] and 0.47% [7], corresponding to one case per 212 and 222 fetuses, respectively. Recently, Annac and Tekin reported an incidence of 0.74% in a group of fetuses who benefited from US in the second and third trimesters, which corresponds to one case for every 135 fetuses [2]. This is not negligible, and the condition is not as uncommon as stated previously. The real incidence of EC remains unknown, and this can only be defined in a large prospective study of non-selected patients in whom careful examination of the fetal gallbladder is made [14]. Our review found an incidence of 0.48%, which is comparable to what previous authors [1,7] have reported.

### 4.2. Terminology

Recent articles [1,35] continue to use the phrase “fetal gallstones,” which we believe does not fully reflect reality and can cause unjustified concern in parents and medical professionals; thus, the terminology of this condition must be revised, as other authors have also suggested [3]. Alternative terms have been proposed for this condition over time: fetal cholelithiasis [4,6,25,31,32,33], echogenic material [3,5,7,20], calcifications [34], and echogenicities [2], which demonstrates the evolution of concept and the recognition that the authors did not agree on a specific name. Since 1992, Brown et al. have shown that we cannot be certain that echogenic material in the fetal gallbladder represents biliary calculi and that only those with distal shadowing may be genuine calculi [5]. Suchet et al. observes in 1993 that the term “cholelithiasis” is questionable in fetuses [15], while Klingensmith and Cioffi-Ragan suggest the name “structures with typical ultrasound findings of gallstone” [10]. Klar et al. offers the interesting terms “biliary pseudolithiasis” and “reversible cholelithiasis” [36]. As the origin and nature of EC remain unknown, we consider it is preferable to provide only the US description of the images instead of a specific condition name, such as “cholelithiasis” or “fetal gallstones”. This aspect requires special consideration because, in the instance of fetal EC, the US description does not guarantee a diagnosis of disease. Informing the mother of a clinical diagnosis based solely on US images could induce an elevated level of anxiety.

### 4.3. Demographic Considerations

It is believed that EC is specific to the third trimester of pregnancy, as only few cases have been documented before 28 weeks, namely at 19 [16], 22 and 24 [32], 26 [30]), and 22 and 24 weeks [2]. It is suggested that if the gallbladder is rigorously evaluated during morphological US in the second trimester, this condition could be diagnosed earlier. However, in the systematic surveillance of 1.133 subsequent high-risk pregnancies assessed before 28 weeks of gestation by Kiserud et al., no cases of EC were detected [20].

According to recent retrospective studies, the median maternal age at the time of EC detection by US was 28 years (range 17–42) [1], 28 ± 4 years (21–40) [2], and 33 years (range 28–39) [3]. Regarding the gender distribution of cases, some authors observe a trend toward a higher prevalence of hyperechogenic foci in male fetuses compared to sludge [3]. In a recent study, 54.6% of fetuses were males and 45.4% were females [2]. An association with male gender has been noted in some series [18], although other studies have not supported male predominance [5,6,31]. Other researchers, however, assert that the incidence of EC is three times higher in female than in male fetuses [30].

Cases of EC in twin pregnancies have been reported in the literature [1,3,5,19,27,29,31,32]. It is possible that only one of the twins, whether they are dizygotic or monozygotic, exhibits this disease [1,3,5,19]. In contrast, both twins presented this condition in a case report [31]. Additionally, the first cases of EC in three siblings are described, which raises the question of whether the mother’s genetics or exposure to environmental factors predisposed to its production [27].

### 4.4. Etiology and Risk Factors

During the fourth week of pregnancy, a ventral outpouching of the caudal region of the embryonic foregut gives rise to the fetal gallbladder [25]. Although a number of authors have attempted to explain the etiology of EC, this issue remains unclear. Various etiopathogenetic theories have been proposed over time and evaluated in published studies [4,5,20], but none has been scientifically confirmed. According to the first theory developed by Brown et al. [5], maternal or placental estrogens may contribute by increasing cholesterol secretion and decreasing bile acid production. If this were the case, the prevalence of EC would likely be higher than reported. Kiserud et al. embraces an ancient theory which proposes that maternal or fetal hemolysis induced by Rhesus or ABO blood group alloimmunization, sickle cell anemia, spherocytosis, or thalassemia may contribute to the development of EC by elevating indirect fetal bilirubin levels [20]. Brown et al. disapproves of this concept [5]. No published article mentions neonatal jaundice, which raises the question of why the affected fetuses are not jaundiced at birth. Decades later, the abovementioned theories remain unproven.

The particularities of bile production, composition, and transit in fetuses could play a role [20]. The absence of regular gallbladder contractions and emptying throughout intrauterine life might predispose to bile stagnation [3], yet other authors have demonstrated that the gallbladder still exhibits contractility [25,37]. The etiopathogenesis of this condition is poorly understood due to the fact that numerous maternal or fetal disorders do not have a common path to EC production. Some of the authors’ assumptions are intriguing and deserving of in-depth study, yet all of them remain simple suppositions.

In recent decades, maternal and fetal predisposing factors have been hypothesized to play a causal role, although none of these theories appear to be supported by evidence [29]. Even in cases with a suggested risk factor, its contribution to the pathophysiology of EC is often difficult to establish [31]. In the reviewed articles, there was no mention of a correlation between genetics and echogenic gallbladder content in fetuses. This could be an intriguing topic for further research.

Nevertheless, considering the known cholestatic effect of ceftriaxone which precipitates insoluble calcium salts, the fact that this antibiotic was administered to the mothers in certain cases of EC is an intriguing observation [1,2]. It is noteworthy that 13 patients (38%) out of 34 pediatric patients between the ages of 3 weeks and 24 months in the study of Klar et al. were treated with third-generation cephalosporins [36]. In a similar manner, progesterone medication to the mother would have the same potential impact of promoting EC development [30].

Two cases who presented with COVID-19 during pregnancy and developed EC were communicated [34,35]. It is unclear whether the recent maternal COVID-19 played a role in the occurrence of these anomalies or if it was simply a coincidence [35]. Currently, it is challenging to establish a causal relationship between the two conditions.

Numerous other maternal risk factors have been proposed, including family history of gallstones, cholecystitis, cholestasis, biliary tree malformation, hepatic dysfunction, obesity, sepsis, methadone use, diabetes, and genetic susceptibility [1,2,3,5,32,35]. The involvement of heredity is an intriguing issue. Cholestasis during pregnancy or a biliary condition (gallstones, cholecystitis, intrahepatic cholestasis of pregnancy) accompanied with dyslipidemia were identified in certain mothers, but a causal association with fetal EC could not be established [1]. Kiserud et al. established a list of conditions associated with EC, including congenital malformations such as tetralogy of Fallot, bilateral clubfoot, and gastroschisis, chromosomal abnormalities (trisomy 21, translocation 10; 11), and extra-amniotic hematoma with intrauterine growth restriction [20].

It is interesting to note that some authors have not identified any fetal risk factors, defining EC as a benign, isolated condition. [5,6,18,22]. Brown et al. shows that the only characteristic common for all twenty-six fetuses in his study was the detection of EC in the third trimester [5]. Cancho Candela et al. conducted a prospective ultrasonographic assessment of 9.235 third-trimester fetuses and found no association with maternal predisposing factors or fetal anomalies [7]. Thus, most cases are considered idiopathic [14,19]. 

### 4.5. Ultrasound Characteristics of EC

Contrary to pediatric and adult patients, EC in the fetal gallbladder might present with a wide variety of US characteristics. Echogenicity, homogeneity, and acoustic artifacts, in addition to the size, shape, and number of echogenic spots, might vary considerably between cases [14,22,31]. Characteristically, there is no dilatation of the biliary tree, pericholecystic fluid, or thickened gallbladder. This allows differential diagnosis with cholesterolosis and adenomyomatosis, two types of hyperplastic cholecystoses characterized by a thick gallbladder wall.

**Figure 2:**
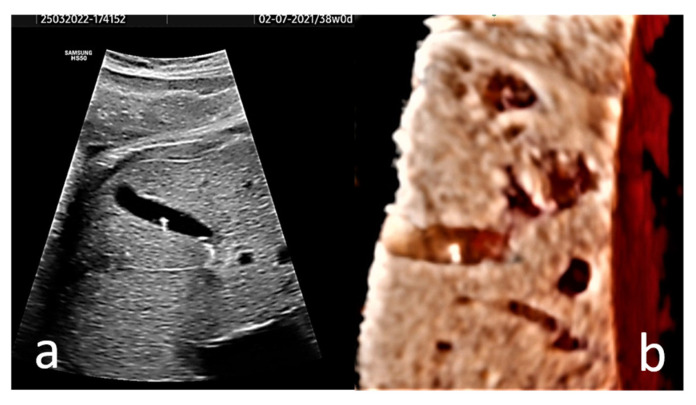
(**a**) Axial section of the gallbladder of a 38 gestational weeks fetus. The hyperechogenic spots are well-defined and do not present distal shadowing; instead they show comet tail artifact. (**b**) A 3D rendered image of the fetus’ gallbladder is provided. A Voluson S8 ultrasound machine with abdominal convex array volume probe RAB6-RS (GE Healthcare, Zipf, Austria) was used.

EC may present in the form of hyperechogenic images, or homogenous echogenic material filling the gallbladder (sludge). Single or multiple hyperechogenic foci are typically confined, well-defined US images with a diameter of less than 1 cm (Figure 2, and Video S1). Due to their shining aspect, they produce a plastic image that we named “starry sky gallbladder” when present in multiples. They may be mobile inside the gallbladder lumen [15,19,27]. We interpret the “mobility” of EC as changes in the position of the hyperechogenic spots between consecutive exams, and not the floating of these structures under the influence of gravitation. The sludge could represent a thickening of the bile or a stage in the spontaneous dissolution of gallstones [6]. It has been stated that 40% of EC manifest sonographically as sludge [3].

Occasionally, the fetal EC may produce acoustic artifacts such as distal shadowing, comet tail, or twinkling [2,4,5]. Annac and Tekin are the only authors to discuss a twinkling artifact, which mimic turbulent blood flow when the reflecting surface is rough [2], and none of the cases in their study presented distal shadowing. In addition, in the research of Sepulveda and Wong, none of the cases showed distal shadowing, indicating that the fetal EC were constituted primarily of cholesterol and were not combined with calcium carbonate or calcium bilirubinate [3]. We support this hypothesis since it is improbable that structures which typically disappear spontaneously without causing symptoms have a composition comparable to that of bones, so they absorb US completely and produce distal shadowing. It is highly probable that the fetal EC occurs in an inhomogeneous liquid environment with zones of varying density. These spots are insufficiently dense to completely absorb US and, like real gallstones, cause a distal shadowing. However, they are sufficiently dense and irregular to generate acoustic anomalies in the form of reverberation or twinkling artifacts. Nevertheless, when observed, distal shadowing is the only finding that can be interpreted as produced by a real stone. When distal shadowing is absent, the EC within the gallbladder can be related to biliary sludge, or hematoma in the gallbladder [15]. Most frequently observed is the comet tail effect, which is a reverberation artifact caused by the interaction of US with small crystals or highly reflective surfaces [2,5]. In other situations, the acoustic artifact is missing, as demonstrated by 35% of cases in a study [5]. Moreover, Brown et al. have shown that the artifact can undergo postnatal modification, as seen in three out of ten cases in their study (in one case, the distal shadowing disappeared postnatally, and in two cases, the comet tail and the distal shadowing, respectively, appeared after birth) [5]. This observation must be regarded with caution, because the performance of US equipment from forty years ago is different from that of modern machines.

It is estimated that over 70% of EC cases are not detected prenatally; therefore, this condition should be included in the genetic sonogram evaluation [30]. According to some authors, polyhydramnios and an enlarged gallbladder frequently manifest at the beginning of the second trimester, prior to the development of EC [17].

In our opinion, the diagnostic value of 3D ultrasonography for fetal EC is reduced since the images provided by several studies [29,30] are unimpressive. Without the need for 3D US imaging, the diagnosis is easy and anatomically correct with 2D US. To illustrate this idea, a 3D US rendered image of the fetus’ gallbladder is provided (Figure 2b).

Other causes of right upper quadrant echogenic images are considered in the differential diagnosis of EC: calcified hepatic masses (hepatoma), gallbladder tumors (hemangioendothelioma, adenoma), intestinal calcifications, and meconial peritonitis. In certain circumstances, such as when the gallbladder is constricted, making a distinction between those conditions can be quite challenging [18,23,27]. Once infection and aneuploidy have been ruled out, the prognosis for isolated hepatic calcifications is favorable, although hepatoma and meconium peritonitis can result in less good outcomes. Therefore, a thorough examination of the fetal abdomen is required to rule out liver foci, hence preventing unnecessary testing and increased maternal anxiety [24,38].

### 4.6. Natural Course

The spontaneous resolution of EC during the first year of life appears to be its natural course. It is commonly believed that EC disappears spontaneously shortly after birth with hydration and feeding [1,27]. Hyperechogenic images and biliary sludge detected during pregnancy appear to have the same resolution time [35].

Numerous hypotheses have been advanced on the dissolution of EC at various moments after birth or even before birth. Beretsky and Lankin indicate that EC formation suggests the possibility of a reversible physiologic situation [4]. The bile flow appears to increase postnatally, and changes in its composition and concentration may contribute to postnatal resolution [1,18]. Sepulveda and Wong hypothesized that once feeding is established after birth, there are higher quantities of cholecystokinin, which increases gallbladder contraction and allows EC to pass into the duodenum [3]. Nevertheless, cases of EC resolution in utero have been documented [2,5,6,7], contradicting the abovementioned theories. An interesting idea suggests that the biliary mucus which produces the EC would be reabsorbed instead of entering the intestine [14]. We agree with this hypothesis and find it counterintuitive that real solid gallstones disintegrate spontaneously after birth or are discharged without symptoms from the biliary ducts. More likely, the EC represent precipitates formed in a highly concentrated bile, and its fluidization after birth due to the onset of oral feeding and the release of digestive fluids causes their dissolution.

Relevant to the elimination of EC could be the US appearance and composition of the stones. Postnatal persistence of EC was observed in 80% of fetuses with distal shadowing, compared to 38% of those with comet tail and 25% of those without artifacts [5]. The composition of EC is unknown because no fetuses or newborns underwent surgical procedures that would permit chemical analysis of the “stones” [2,3]. It was hypothesized that they are composed of cholesterol, calcium crystals, and pigment [1,14]. To our knowledge, however, no prenatally identified calculi have been analyzed, and pathological confirmation is lacking. It is conceivable that mucus is the cause of the fetal EC [14]. Schirmer et al. observed that none of the calcified (radiopaque) stones disappeared spontaneously after birth, unlike the cholesterol, urate, and pigment stones (nonradiopaque) [11].

When developing criteria for the severity of prenatal EC, early gestational age and underlying familial or maternal conditions must be taken into account. In one of the reported cases, the presence of biliary sludge before 26 weeks of gestation was a determinant of neonatal severity and chronicity [30]. If biliary sludge emerges before 26–24 weeks of gestation, Caroli’s syndrome and epidermal nevus syndromes may be the cause [30,39]. In contrast, the development of EC after 30 weeks of gestation in the absence of risk factors was associated with a favorable prenatal and neonatal clinical course [30].

### 4.7. Complications

The EC is regarded as benign and transient in fetuses [2,31], in contrast with cases of gallstones in children and adults where spontaneous resolution is uncommon and surgery is frequently necessary. In the research cited, complications such as biliary dilatation, intra-abdominal ascites, jaundice, and feeding difficulties are rare or absent. Schwab et al. followed a subset of 17 patients for 3–20 years and found no complications or long-term sequelae associated with fetal gallstones [1]. In a recent study, Annac and Tekin followed 43 of 44 newborns with EC up to 1 year of age, and none of them developed gastrointestinal or hepatic disorders during the course of the study [2]. In all cases reported by Sepulveda and Wong, complications were nonexistent [3]. However, some authors believe that caution is required because prenatal EC should be regarded as an indicator of severe fetal malformation or disease in at least 20% of cases [30]. In the study conducted by Brown et al., seventeen patients were followed up; in nine cases, resolution was noted, whereas three cases exhibited persistent EC at 4.5 years [5]. A patient with biliary symptoms and persistent EC at 2.5 years is documented [30]. At autopsy, a reticulonodular liver and an enlarged gallbladder with multiple gallstones were found in a hydropic fetus, which suggests that EC may be a sonographic sign linked with hemochromatosis [40]. We must be aware of the fact that a number of patients were not followed up in the reviewed studies, and their subsequent evolution is uncertain.

The prognosis for EC is regarded to be favorable [2,3,7]. However, there are authors that challenge this assertion. Approximately 11% of cases of prenatal EC precede cholelithiasis and cholecystitis in children and adolescents, as well as hepatobiliary illness in adults [30]. Chronic prenatal cholelithiasis or biliary sludge beyond one year of age may predict a poorer prognosis, as illustrated by the case of a 16-day-old adolescent who required cholecystectomy due to chronic cholelithiasis [41]. The need for cholecystectomy was reported in two cases at 11 and 14 years of age due to cholecystitis associated with long-term persistence of gallstones that had been detected prenatally [30].

Given the rarity of cases and the paucity of studies, there are no recommendations for best practice in the situation of EC. Authors advocate that these cases be referred to a maternal fetal medicine unit for differential diagnosis and exclusion of structural defects or hereditary conditions [20,30,42]. Additional prenatal scans are not required since obstetric care or birth will remain unchanged [17,25,35]. Except for the twins, all fetuses in the study by Sepulveda and Wong were delivered at full term [3]. Term births occurred either naturally or through caesarean section according to obstetric indications. EC is not an indication of preterm birth or caesarean section in itself.

### 4.8. Follow-Up

A postnatal US is required to determine the number and aspect of the EC and rule out obstruction or abnormalities of the biliary tract [24,28]. Due to the high possibility of spontaneous resolution, the suggested approach is to reassure parents and follow the evolution without therapy, but with periodic US scans until resolution or during the first year of life [29,30]. Tonni and Ruano suggests performing close prenatal and postnatal monitoring with postnatal laboratory tests (liver function and enzymes) and abdominal ultrasound [42]. On the basis of their data, other authors do not propose postnatal imaging in asymptomatic patients [1,2]. In the study by Sepulveda and Wong, postnatal abdominal ultrasonography was performed in four cases, primarily due to parental anxiety, and all of the results were normal [3].

Two authors [24,27] advocate the use of ursodeoxycholic acid in newborns. In one study, two siblings received ursodeoxycholic acid at a dosage of 15 mg/kg per day, divided into two doses, for eleven months and five weeks, respectively [27]. In other research, two babies were initiated on ursodeoxycholic acid at a dosage of 5–7 mg/kg/day two weeks after birth, but the duration of treatment is undisclosed [24]. Other authors consider that medical treatment or cholecystectomy should be considered for extremely rare cases with jaundice or biliary obstruction [28,35,36]. Hurni et al. report a 36-year-old woman with a monochorionic diamniotic twin pregnancy who was diagnosed with intrahepatic cholestasis of pregnancy. From 32 weeks of gestation, the patient was treated with a combination of ursodeoxycholic acid, levocetirizine, and cholestyramine, but the dosages are not mentioned in the article. At 33 weeks, both twins were diagnosed with intra-cystic cholelithiasis [31].

**Figure 3:**
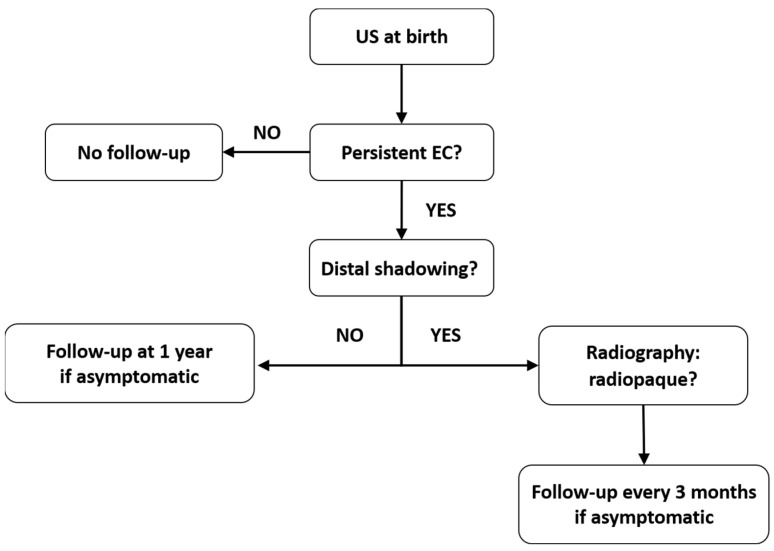
Follow-up algorithm suggested for patients with prenatal EC in the gallbladder.

There are currently no established protocols for the follow-up of asymptomatic patients, which has resulted in a paucity of long-term outcome data [1]. However, there are cases with persistent EC that require follow-up until resolution to eliminate potential complications. Taking into account the current understanding of EC, we propose a follow-up plan for these cases (Figure 3). We considered the data regarding the US aspect, composition, and natural history of EC, as summarized from the available studies.

### 4.9. Counseling

Parents must be informed that fetal EC is frequently associated with a favorable prognosis and spontaneous postnatal resolution, and that scientific evidence does not warrant medical or surgical intervention in asymptomatic patients [40]. We concur with the suggestion made by Hurni et al. [31] that clinical and ultrasonographic monitoring must continue until resolution is evident.

### 4.10. Limitations

As prenatal detection of EC is uncommon, this study is limited by the retrospective nature of the studies and the small number of cases reported in the literature. In addition, the ultrasound features of fetal EC and the outcome of the babies at a specific time after birth are not clearly defined in some articles. The fact that a number of patients are lost to follow-up is another limitation of our research.

### 4.11. Future Research Directions

Given the number of cases with EC which are not detected prenatally, it is worthwhile to include the gallbladder in the third trimester ultrasound assessment of fetal anatomy. To elucidate aspects of the etiology, natural course, and outcome of this condition, further prospective cohort studies are necessary. The contribution of genetic abnormalities in the development of EC would offer a fascinating research topic.

## 5. Conclusions

Commonly, EC is detected during the third trimester of pregnancy. It is highly probable that many cases are not detected until after birth; consequently, fetal gallbladder examination should be included in the third trimester US protocol. In the vast majority of cases, the condition is benign and self-limiting, since it resolves spontaneously throughout the postnatal period. However, it is essential to distinguish it from other echogenic images in the upper quadrant of the abdomen, as a number of them are associated with poor prognosis. After adequately reassuring the parents, the patients should be followed-up for spontaneous resolution, with no medical or surgical intervention recommended. Patients with EC who are asymptomatic can be managed with a wait-and-see attitude. Unknown is the future risk of developing gallstones. Since evolution might differ from case to case, it is necessary to personalize the management of EC, as the optimal timing for follow-up has not yet been determined. Our suggestion for the management of these patients is based on the current knowledge of the features and natural history of EC.

## Figures and Tables

**Table 1:** Summary of the studies under review.

First Author	Year	Study Type	Term	Period	n Scans	n Cases
Beretsky [4]	1983	case report	cholelithiasis			1
Heijne [9]	1985	case report	gallstones			1
Klingensmith [10]	1988	case report	gallstones			1
Schirmer [11]	1989	case report	cholelithiasis			1
Abbitt [12]	1990	case report	gallstones			1
Broussin [13]	1990	case report	cholelithiasis			3
Devonald [14]	1992	retrospective	gallstones	1990	1.104	7
Brown [5]	1992	retrospective/prospective	echogenic material	1984–1991		17/9
Suchet [15]	1993	case report	cholelithiasis			1
Clarke [16]	1994	case report	cholelithiasis			1
Petrikovsky [17]	1996	case report	sludge			5
Stringer [18]	1996	case report	gallstones			3
Sepulveda [19]	1996	case report	echogenic material			1
Kiserud [20]	1997	retrospective	echogenic material		1.656	6
Nishi [21]	1997	case report	cholelithiasis			1
Suma [22]	1998	case report	gallstones			2
Hertzberg [23]	1998	case report	gallstones			2
Agnifili [6]	1999	prospective	cholelithiasis	1995–1996	764	3
Cancho Candela [7]	2004	prospective	echogenic material		9.235	42
Munjuluri [24]	2005	case report	gallstones		3.015	2
Lariviere [25]	2006	case report	cholelithiasis			1
Sheiner [26]	2006	case report	gallstones			4
Iroh Tam [27]	2010	case report	gallstones			2
Holloway [28]	2010	case report	cholelithiasis			1
Triunfo [29]	2013	case report	cholelithiasis			1
Troyano-Luque [30]	2014	case report	cholelithiasis			2
Hurni [31]	2017	case report	cholelithiasis			2
Kesrouani [32]	2018	case report	cholelithiasis			3
Gomez [33]	2018	case report	cholelithiasis			1
Sepulveda [3]	2020	retrospective	echogenic material	2014–2017	4.026	19
Sileo [34]	2021	case report	calcifications			1
Kahlon [35]	2021	case report	gallstones			1
Annac [2]	2021	retrospective	echogenicities	2015–2020	5.977	44
Schwab [1]	2022	retrospective	gallstones	1996–2019		34
					**TOTAL**	**226**

**Table 2:** Ultrasound features of the echogenic content detected in the fetal gallbladder.

First Author	*n*	US Aspect			US Artifact			
		Sludge	Single Focus	Multiple Foci	Shadowing	Comet Tail	Twinkling	No Artifact
Beretsky [4]	1			1	1			
Heijne [9]	1			1		n/a		
Klingensmith [10]	1			1	1			
Schirmer [11]	1			1		n/a		
Abbitt [12]	1			1				1
Broussin [13]	3			3		n/a		
Devonald [14]	7	1	1	5	1			6
Brown [5]	26	4 (15%)	3 (12%)	19 (73%)	8 (30%)	9 (35%)		9 (35%)
Suchet [15]	1			1				1
Clarke [16]	1		1			n/a		
Petrikovsky [17]	5	5				n/a		
Stringer [18]	3			3		n/a		
Sepulveda [19]	1	1				n/a		
Kiserud [20]	6	2		4		n/a		
Nishi [21]	1		1					1
Suma [22]	2		1	1	1			
Hertzberg [23]	2			2	1			
Agnifili [6]	3	1		2				3
Cancho Candela [7]	42	31 (74%)	4 (9%)	7 (17%)		n/a		
Munjuluri [24]	2		1	1				2
Lariviere [25]	1			1		n/a		
Sheiner [26]	4			4		n/a		
Iroh Tam [27]	2	1		1		n/a		
Holloway [28]	1		1			n/a		
Triunfo [29]	1			1				1
Troyano-Luque [30]	2	1		1				1
Hurni [31]	2			2				1
Kesrouani [32]	3			3		n/a		
Gomez [33]	1		1		1			
Sepulveda [3]	19	8 (42%)	3 (16%)	8 (42%)	0	n/a		
Sileo [34]	1		n/a			n/a		
Kahlon [35]	1			1		n/a		
Annac [2]	44	10 (22.7%)	9 (20.4%)	25 (56.8%)	n/a	28 (63.6%)		
Schwab [1]	34	7 (20.6%)	4 (11.8%)	n/a	2 (5.4%)	n/a		
**TOTAL**	226	72	30	100	16	37		26

n/a: not available.

**Table 3:** Outcomes of newborns with prenatally detected echogenic gallbladder content.

	First Author	*n*		Resolution		Persistent	Remarks
			Before Birth	at Birth	<1 Year		
1	Beretsky [4]	1			1		
2	Heijne [9]	1			1		
3	Klingensmith [10]	1			1		
4	Schirmer [11]	1		n/a			asymptomatic at 2 y
5	Abbitt [12]	1			1		
6	Broussin [13]	3			2		1 lost to f/u
7	Devonald [14]	7			5	1	1 lost to f/u
8	Brown [5]	26	2		7	3	17/26 postnatal imaging; resolution in 9/17 (53%); 1 demise after birth
9	Suchet [15]	1			1		
10	Clarke [16]	1			1		
11	Petrikovsky [17]	5			4		1 refuses study
12	Stringer [18]	3			2	1	
13	Sepulveda [19]	1		1			
14	Kiserud [20]	6		2	3	1	
15	Nishi [21]	1		1			
16	Suma [22]	2			2		
17	Hertzberg [23]	2		1	1		
18	Agnifili [6]	3	1		2		
19	Cancho Candela [7]	42	1		38		3 lost to f/u; 1 demise after birth
20	Munjuluri [24]	2			2		
21	Lariviere [25]	1					no f/u
22	Sheiner [26]	4			4		
23	Iroh Tam [27]	2			2		
24	Holloway [28]	1				1	
25	Triunfo [29]	1		1			
26	Troyano-Luque [30]	2			1	1	symptomatic
27	Hurni [31]	2			2		
28	Kesrouani [32]	3				1	1 asymptomatic; 1 lost to f/u
29	Gomez [33]	1				1	
30	Sepulveda [3]	19		2	13		4 n/a
31	Sileo [34]	1		1			
32	Kahlon [35]	1			1		
33	Annac [2]	44	5		38		38/44 with f/u; 1 lost to f/u
34	Schwab [1]	34			3	1	8/34 with postnatal imaging (1–18 years); 17 with f/u (3–20 years); 1 demise in utero
	**TOTAL**	**226**	**9**	**9**	**138**	**6 > 1 year**	

f/u: follow-up; n/a: not available.

## Data Availability

Not applicable.

## Supplementary Materials

The following supporting information can be downloaded at: https://www.mdpi.com/article/10.3390/diagnostics13020230/s1, Video S1: Echogenic content with “comet tail” artifacts in the gallbladder of a 38 gestational weeks fetus.
